# Letter from Dr. S. C. Harbert

**Published:** 1848-01

**Authors:** S. C. Harbert


### ARTICLE VII.

### *Letter from* Dr. S. C. Harbert.

Messrs. Editors :

In the October number of the "Journal," I find over the signature of S. P. Hullihen, an article which, I suppose the author would leave its readers to regard, as a *fair* and *impartial* review of my work on Surgical and Mechanical Dentistry; it is not my purpose in replying to Mr. H. to adopt a tone and character so familiar to him throughout, but "by every *word, deed and action*," to maintain a *consistency* and *truthfulness*, that should belong to every member of the profession.

In the *"review"* alluded to, the writer, after spending his ammunition upon the preface, proceeds to an attack upon the innocent bookbinder, for having placed the plates, "between the index and preface of the book; and then, he attempts an analysis and history of the plates. The figures on plate 1, he says, were copied "from the works of Dr. Paré" and "from a German work, before the time of Paré." This charge is false in every particular, nor do I believe Mr. H. possessed the slightest foundation for such a supposition. If he will refer to page 83 of my work, he will there learn that no merit to originality is claimed in the keys; the excavators as well as the keys, were *selected* from my own case, as considered the most desirable for practical use, the latter (particularly) from their simplicity.

His descriptions of plates 2, 3 and 4, are but as daguerreotypes of the whole, the gums "of brick-dust hue" are as gratuitous, as the "beeswax" his excited imagination has placed upon the plaster cast; while such as afford no possible means for his *deductive* mind, to animadvert upon, is represented as useless, or of "doubtful qualifications."

As to the claims of the profession to the signature of "art" or science. I will not here discuss, though I do not see the applicability of the latter, to the present coherent state of dental practice. Of the fourth page of the "*review*," one half is occupied in quotations, wherein a single error in phraseology, is made to sustain its share, in supporting a criticism, "*fully, fairly* and *fearlessly*" executed. Then follows, an error and neglect, which in justice to the Cincinnati College, would have been made in an "errata" in the back part of the book, but to conciliate the printer's views and convenience; other occasional errors were suffered to pass unnoticed from a like cause. The word "Cincinnati" was in the manuscript, with "Philadelpia" interlined, the first was omitted and the latter inserted. Mr. H. is mistaken as to my place of residence; I was obliged from ill health, six years ago, to remove from the city, and have ever since that time, been living sixty miles distant; my distance precluded the possibility of examining every proof sheet, before it went to press. The Philadelphia institution, I was informed by a friend, was designed to be collegiate. On the subsequent page of the review, a passage (in the book) clearly marked as a quotation, is given as original, evidently with the design of creating a false impression with those to whom it may be familiar, and in his remarks upon the "treatment of caries," an attempt is made by placing in juxtaposition, isolated sentences, to prove an inconsistent practice. In conclusion, no man, in putting forth a work upon any subject, can expect to meet with universal favor, nor will a community suffer themselves to be gulled by every garbled statement, that emanates from the caput of any obscure critic, whose sole purpose, would seem by displaying himself before the world, as an able and honest reviewer, to build up his reputation, by the sacrifice of another's.

With the candid of the profession, I leave it to judge, whether the practice, as recommended in my work is erroneous or correct; that I have not been as full as the various subjects will admit of, I have not the presumption to suppose; in the desire to comprehend the leading branches of the practical dentist, in a limited space, much must necessarily be omitted, of service to the accomplished practitioner.

[ As Mr. Harbert seems to think that injustice has been done him in the review of his work which appeared in the last number of the Journal, we cheerfully insert the foregoing defence, but we cannot perceive that he has placed his work in a less exeptionable light, or pointed out wherein he had been misrepresented. The mere fact that one of the engravings, of his work represents instruments which he has in his possession, is no proof that Paré's work does not contain engravings of them; equally has he failed to establish the consistency of the doctrine which he has laid down; we cannot see, therefore, that the reviewer has dealt unfairly with him. With Mr. Harbert personally, we know nothing, never having heard his name, to the best of our recollection, until after the publication of his work, and should be very sorry to have him misrepresented through the pages of the Journal, and from the well known reputation of Dr. Hullihen, not only as one of our most scientific and skilful practitioners, but as an honorable highminded man, we feel sure he had no such intention.]—*Balt. Ed.*

